# Diet Quality and Dietary Inflammatory Index in Dutch Inflammatory Bowel Disease and Irritable Bowel Syndrome Patients

**DOI:** 10.3390/nu14091945

**Published:** 2022-05-06

**Authors:** Marlijne C. G. de Graaf, Corinne E. G. M. Spooren, Evelien M. B. Hendrix, Martine A. M. Hesselink, Edith J. M. Feskens, Agnieszka Smolinska, Daniel Keszthelyi, Marieke J. Pierik, Zlatan Mujagic, Daisy M. A. E. Jonkers

**Affiliations:** 1 Division Gastroenterology-Hepatology, Department of Internal Medicine, Maastricht University Medical Center+, P.O. Box 5800, 6202 AZ Maastricht, The Netherlands; c.spooren@maastrichtuniversity.nl (C.E.G.M.S.); e.hendrix@maastrichtuniversity.nl (E.M.B.H.); martine.hesselink@maastrichtuniversity.nl (M.A.M.H.); daniel.keszthelyi@maastrichtuniversity.nl (D.K.); m.pierik@mumc.nl (M.J.P.); z.mujagic@maastrichtuniversity.nl (Z.M.); d.jonkers@maastrichtuniversity.nl (D.M.A.E.J.); 2 NUTRIM School of Nutrition and Translational Research in Metabolism, Faculty of Health, Medicine and Life Sciences, Maastricht University, P.O. Box 616, 6200 MD Maastricht, The Netherlands; a.smolinska@maastrichtuniversity.nl; 3 Division of Human Nutrition and Health, Department of Agrotechnology and Food Sciences, Wageningen University & Research, P.O. Box 17, 6700 AA Wageningen, The Netherlands; edith.feskens@wur.nl; 4 Department of Pharmacology and Toxicology, Maastricht University, P.O. Box 616, 6200 MD Maastricht, The Netherlands

**Keywords:** Dutch Healthy Diet Index 2015, Adapted Dietary Inflammatory Index, gastrointestinal disease, intestinal inflammation, gastrointestinal symptoms

## Abstract

Inflammatory bowel disease (IBD) and irritable bowel syndrome (IBS) share common culprit foods and potential pathophysiological factors. However, how diet may contribute to disease course and whether this differs between both entities is unclear. We therefore investigated the association of dietary indices with intestinal inflammation and gastrointestinal symptoms in both IBD and IBS patients. Food frequency questionnaires from 238 IBD, 261 IBS and 195 healthy controls (HC) were available to calculate the overall diet quality by the Dutch Healthy Diet-Index 2015 (DHD-2015) and its inflammatory potential by the Adapted Dietary Inflammatory Index (ADII). Intestinal inflammation and symptoms were evaluated by faecal calprotectin and the Gastrointestinal Symptom Rating Scale, respectively. The DHD-2015 was lower in IBD and IBS versus HC (*p* < 0.001), being associated with calprotectin levels in IBD (b = −4.009, *p* = 0.006), and with abdominal pain (b = −0.012, *p* = 0.023) and reflux syndrome (b = −0.016, *p* = 0.004) in IBS. ADII scores were comparable between groups and were only associated with abdominal pain in IBD (b = 0.194, *p* = 0.004). In this side-by-side comparison, we found a lower diet quality that was differentially associated with disease characteristics in IBD versus IBS patients. Longitudinal studies are needed to further investigate the role of dietary factors in the development of flares and predominant symptoms.

## 1. Introduction

Inflammatory bowel disease (IBD) and irritable bowel syndrome (IBS) are both multifactorial and heterogeneous intestinal disorders. IBD is a chronic inflammatory disease, comprising Crohn’s disease (CD) and ulcerative colitis (UC), and is characterised by alternating sequences of active inflammation and remission. IBD is generally considered to arise from a complex interaction between host genetics, the intestinal microbiome and immune factors, as well as environmental factors [1,2]. The latter is supported amongst others by the rising incidence in line with Westernisation [3]. IBS is found to be present in 5–10% of the Western population [4], and is characterised by recurrent abdominal pain in combination with altered bowel habits. In addition to microbiome perturbations, alterations in intestinal motility, barrier function, visceral perception and brain–gut interaction, a low-grade inflammation is reported in subgroups of IBS patients. Although the exact underlying mechanisms are not clear, symptoms can also be triggered by environmental factors [5]. IBS-like symptoms are also reported in about 35% of IBD patients in remission [6].

One of the environmental factors associated with both IBD and IBS is the Western diet, characterised by, for example, high fat, high sugar and low fruit and vegetable intake [7,8]. Furthermore, 58–68% of IBD patients with active disease, 29–39% of IBD patients in remission [9] and up to 90% of IBS patients [10] indicate that meals and/or certain food products exacerbate flares and/or gastrointestinal (GI) symptoms. Dairy products, spicy foods, wheat products and gas-producing foods including some fruits and vegetables, are reported to be the main culprits by both patient groups [9,10]. Diet can influence both disease onset and disease course, for example, through interaction with the immune system, but also by modulating the intestinal microbiota composition and activity and/or intestinal barrier function [7,8].

As a consequence, interest is increasing in nutrients or foods that have an (anti-)inflammatory potential or can contribute to GI symptoms, for example, by increased gas production and osmotic effects [7,8]. As foods are generally not consumed in isolation, but as part of the total diet, this further adds to the complexity. Although various dietary intervention strategies are currently being investigated, it is not completely clear how overall diet quality in IBD and IBS relates to inflammation markers and symptom occurrence.

Various indices have been developed to assess diet quality. Overall diet quality can be defined by adherence to the Dutch dietary guidelines [11] by calculating the Dutch Healthy Diet Index 2015 (DHD-2015) [12]. Furthermore, a diet can be defined by its pro- or anti-inflammatory potential, and by calculating indices based on the (anti-)inflammatory properties of certain nutrients and food items. Examples of these indices include the Adapted Dietary Inflammatory Index (ADII), based on nutrients [13]; and the Empirical Dietary Inflammatory Index (EDII), based on food products [14].

IBD and IBS share common culprit foods as well as underlying mechanisms, but the magnitude of these factors differs between the diseases, for example, with inflammation being more prominent in IBD. Therefore, a side-by-side comparison of IBD and IBS can provide further insight into the association of overall diet quality with markers for inflammation and symptom occurrence. This may identify leads for further mechanistic studies and will aid in providing patients with adequate advice. Therefore, we aim to investigate the relationship of the adherence to the Dutch dietary guidelines (using the DHD-2015) and the inflammatory potential of the diet (using the ADII) with inflammatory markers and GI symptoms in both IBD and IBS patients.

## 2. Materials and Methods

### 2.1. Study Population

For this study, cross-sectional data on habitual dietary intake and clinical data were collected from two large cohorts from the same geographical region in the Netherlands. All participants provided written informed consent prior to participation.

#### 2.1.1. IBD South Limburg Cohort

The IBD South Limburg (IBDSL) cohort is a well-characterised population-based inception cohort in the South Limburg area in the Netherlands and has been used to study IBD epidemiology and disease course since 1991 [15]. Patients included were at least 18 years old and were diagnosed with either CD or UC according to the Lennard-Jones criteria [16] and proven by endoscopic, radiological and/or histological findings. Relevant demographical and clinical data were retrieved from the IBDSL data warehouse [15]. Data on habitual dietary intake were collected using a validated food frequency questionnaire (FFQ) as part of a sub-study within the IBDSL cohort. Both the IBDSL cohort and the sub-study have been approved by the medical research ethics committee of the Maastricht University Medical Center+ (MUMC+) (NL31636.068.10 and NL42101.068.12, respectively), and have been registered at the US National Library of Medicine (NCT02130349 and NCT0176963, respectively).

#### 2.1.2. Maastricht IBS Cohort

The Maastricht IBS (MIBS) cohort has been used to study the phenotypical and geno-typical characterisation of patients with IBS at the MUMC+ since 2009. All patients included were at least 18 years old and complied with the Rome III criteria for IBS [17]. Furthermore, healthy controls (HC) were included as described previously [18]. The MIBS cohort was approved by the medical research ethics committee of the MUMC+ (NL24160.068.08) and has been registered at the US National Library of Medicine (NCT00775060). Participants with dietary intake data as part of a previous study [19] were re-analysed for the current study.

### 2.2. Demographic and Clinical Data Collection

In both cohorts, demographic and clinical characteristics were collected including age, sex, Body Mass Index (BMI), smoking, medication use and disease phenotype. Faecal calprotectin was used as the marker for intestinal inflammation. Faecal samples were collected at home, stored in a fridge and brought to the hospital within 24 h after defecation for routine analysis of faecal calprotectin by the clinical chemistry department using a fluorescent enzyme immune assay (FEIA) (IBDSL cohort), or using a commercial enzyme-linked immunosorbent assay (ELISA, Bühlmann Laboratories, Schönenbuch, Switzerland) (MIBS cohort). The presence of GI symptoms was assessed using the Gastrointestinal Symptom Rating Scale (GSRS), consisting of 16 items clustered into five major GI syndromes: abdominal pain, reflux syndrome, diarrhoea syndrome, indigestion syndrome and constipation syndrome [20].

For IBD patients, disease phenotype at time of inclusion was defined by the Montreal classification, including age of onset, disease location and behaviour (for CD), or extent (for UC) [21]. Furthermore, disease duration, clinical activity indices (i.e., Harvey Bradshaw Index (HBI) for CD [22] and Simple Clinical Colitis Activity Index (SCCAI) [23] for UC) and time since last flare were retrieved from the IBDSL data warehouse. A flare was defined by the following criteria, in line with clinical practice and previous studies [24,25]: (1) presence of active disease confirmed by a physician based on endoscopy and/or radiological imaging; (2) faecal calprotectin ≥ 250 μg/g; (3) faecal calprotectin ≥ 100 μg/g with at least a five-fold increase from previous visit; (4) clinical symptoms indicative for active disease or increased HBI (≥5) or SCCAI (≥3) accompanied by dose escalation or initiation of a new drug; or (5) dose escalation or initiation of a new drug accompanied by C-reactive protein (CRP) ≥ 10 mg/L. Active disease at inclusion was defined as having a flare at inclusion or during the three months prior to inclusion. In addition, when data were incompletely registered in patients’ records in the period before inclusion, IBD-related hospitalisation due to disease activity and IBD-related surgery were examined to be able to evaluate disease activity.

For IBS patients, subtypes—diarrhoea (IBS-D), constipation (IBS-C), mixed stool pattern (IBS-M) and unspecified stool pattern (IBS-U)—were defined according to the Rome III criteria [17].

### 2.3. Dietary Data Collection

Habitual dietary intake was evaluated by using the same self-administered FFQ in both cohorts, with a recall period of a month, which has been developed and validated by the division of Human Nutrition of Wageningen University [26,27]. The intake was assessed by scoring the frequency of consumption and by estimating portion sizes using natural portions and commonly used household measures. The intake of nutritional supplements was not included in the FFQ; it was recorded separately. Data were linked to the Dutch food composition table (NEVO 2010, RIVM, Bilthoven, the Netherlands), resulting in a calculated individual mean consumption of 45 nutrients and 148 food items.

Only participants with complete dietary intake, clinical and demographic data were eligible for inclusion in the current study. Participants were excluded if they were on tube feeding or if FFQ data were incomplete or considered implausible, i.e., an overall intake for males <800 or >4000 kcal/day and for females <500 or >3500 kcal/day [28].

#### 2.3.1. Dutch Healthy Diet Index 2015 (DHD-2015)

To assess the adherence to the Dutch healthy diet guidelines [11], the DHD-2015 was computed as described previously by Looman et al. [12]. Based on our FFQ data, the difference between filtered and unfiltered coffee could not be made, and salt intake could not be calculated, finally resulting in 13 components available for our calculation (Appendix A, Table A1 and Table A2). Briefly, for each component a minimum, maximum or optimum intake was defined. Based on these criteria, each component received 0–10 points, resulting in a total score ranging from 0 to 130 points. A higher score indicates a better adherence to the dietary guidelines.

#### 2.3.2. Adapted Dietary Inflammatory Index (ADII)

To assess the inflammatory potential of the diet, the ADII was computed as described previously by Van Woudenberg et al. [13]. The ADII is a literature-derived index that summarises an individual’s diet on the continuum from maximally anti-inflammatory to maximally pro-inflammatory. The score was defined by the pro- or anti-inflammatory properties of various macro- and micronutrients based on a literature search for their effect on inflammatory markers (i.e., IL-1β, IL-4, IL-6, IL-10, TNF–α and CRP). This resulted in a (weighed) positive (pro-inflammatory) or a negative (anti-inflammatory) value for each component. The sum finally indicates the overall diet score, which has been validated in healthy individuals, elderly and those at risk of type 2 diabetes and cardiovascular disease [13,29,30], and used in various patient groups [31,32,33,34].

Based on our FFQ data, the exact intake of caffeine, quercetin and garlic could not be calculated, resulting in 26 components available for our calculation (Appendix A, Table A3). First, the intake of each component was adjusted for energy-intake using the residual method. As energy intake was significantly different between groups, the ADII was computed separately for IBD, IBS and HC. Next, this calculated standardised energy-adjusted intake was multiplied by the inflammatory weight. Then, these values were summed to obtain the final score. A higher (positive) score points to a more pro-inflammatory diet, whereas a lower (negative) score indicates a more anti-inflammatory diet.

### 2.4. Statistical Analyses

A statistical analysis was performed using IBM SPSS Statistics version 26.0 [35]. Normality of data was checked using a normal probability plot. Baseline characteristics were presented as mean with corresponding standard deviation (SD) for continuous parametric variables, and as percentages for categorical variables. Differences in baseline characteristics between IBD patients, IBS patients and HC were tested with an analysis of variance (ANOVA) and post-hoc Bonferroni correction (for continuous data) and the Chi-square test with Fisher exact when necessary (for categorical data).

A linear regression analysis was used to assess the association between the dietary indices (DHD-2015 or ADII) and intestinal inflammation (using faecal calprotectin as marker) or GSRS domains. Analyses were performed for each subgroup (IBD, IBS, HC) separately. The following parameters were included in the analyses: age, sex, smoking, BMI, medication, subtype (IBS) or phenotype (IBD), and for IBD patients, additionally, disease duration (in years) and age at diagnosis (defined by the Montreal classification). Missing values were excluded listwise. A two-sided *p*-value < 0.05 was considered to be statistically significant.

In addition to using predefined indices (i.e., DHD-2015 and ADII), an explorative unsupervised random forest (URF) analysis [36] was performed to identify possible combinations of food items or nutrients of relevance to distinguish IBD, IBS and HC. More details can be found in Appendix B.

## 3. Results

### 3.1. Baseline Characteristics

Complete FFQ data were available for 239 IBD patients, 274 IBS patients and 207 HC. Because of implausible low or high intake, 1 IBD patient, 13 IBS patients and 12 HC were excluded, resulting in 238 IBD patients, 261 IBS patients and 195 HC being included in the present study.

Demographic and clinical characteristics are displayed in Table 1. Age was comparable between IBD patients (45.7 ± 14.8 years), IBS patients (43.3 ± 17.0 years) and HC (44.4 ± 18.9 years). In the IBS group, significantly more women (74%) were included as compared to IBD (52.9%, *p* < 0.001) and HC (63.1%, *p* = 0.007). BMI was significantly lower in HC (23.9 ± 3.8 kg/m^2^) compared to IBD (25.5 ± 4.2 kg/m^2^, *p* < 0.001) and IBS patients (25.0 ± 4.6 kg/m^2^, *p* = 0.021). Smoking behaviour was also significantly different between groups, with more active smokers in IBD (20.4%, *p* < 0.001) and IBS patients (23.6%, *p* < 0.001) as compared to HCs (6.7%), and more former smokers among the IBD patients (41.7%) compared to IBS (24.4%, *p* < 0.001) and HC (31.8%, *p* = 0.035).

The IBD patients comprised of 156 CD (65.5%) and 82 UC (34.5%) patients, with 61.5% of all patients (36.5% and 28.0%, respectively) being in remission at the time of inclusion. In IBS patients, the IBS-M subtype was predominant (39.5%), followed by IBS-D (35.6%), IBS-C (21.5%) and IBS-U (3.4%).

### 3.2. Dietary Intake, Diet Quality and Inflammatory Potential of the Diet

Mean total energy intake was significantly lower in IBS (1939.6 ± 604.9 kcal) when compared to IBD (2180.0 ± 634.3 kcal, *p* < 0.001) and HC (2180.4 ± 622.9, *p* < 0.001). Full details on the intake of specific food items and nutrients are given in Appendix A, and Table A2 and Table A3, respectively.

**Figure 1:**
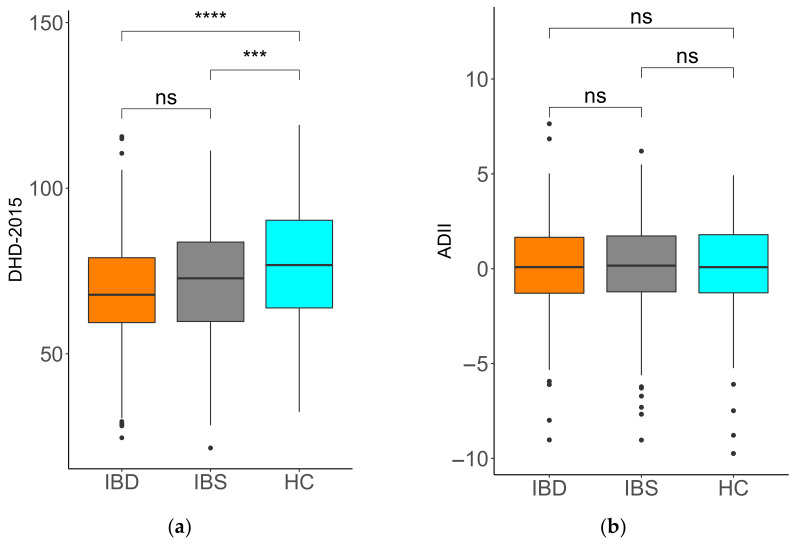
Dietary indices for inflammatory bowel disease (IBD), irritable bowel syndrome (IBS) and healthy controls (HC). (**a**) Dutch Healthy Diet Index 2015 (DHD-2015), (**b**) Adapted Dietary Inflammatory Index (ADII). The difference between subgroups was tested with analysis of variance (ANOVA) and post-hoc Bonferroni correction. ns = not significant, *** *p* < 0.001 and **** *p* < 0.0001.

The DHD-2015 (Figure 1a) ranged from 24.64 to 115.58 in IBD, 21.57–111.34 in IBS and 32.47–119.10 in HC, with a significantly lower mean in IBD (69.00 ± 16.53) and IBS (71.61 ± 16.58) as compared to HC (77.34 ± 17.43; IBD vs. HC: *p* < 0.001; IBS vs. HC: *p* = 0.001; IBD vs. IBS: *p* = 0.251).

For all groups, adherence to the Dutch dietary guidelines was highest for alcohol, whole grain and red meat. However, the absolute intake of vegetables, fruit, whole grain products and the DHD-2015 score for these components were significantly lower in IBD and IBS as compared to HC. Furthermore, in both IBD and IBS, the absolute intake for dairy was significantly lower as compared to HC, but this did not reflect in a significantly lower DHD-2015 score. In IBD only, absolute intake of red meat was significantly higher compared to IBS and HC; this reflected in a significantly lower DHD-2015 score for this component. The lowest mean component scores were observed for refined grain, nuts, and processed meat (for IBD and IBS) or tea (for HC). The exact order of highest and lowest component scores was slightly different per subgroup (Appendix A, Table A2).

The ADII scores (Figure 1b) ranged from −9.02 to 7.64 in IBD, from −9.03 to 6.20 in IBS and −9.74–4.93 in HC, with a mean score that did not differ between IBD (0.052 ± 2.41), IBS (0.055 ± 2.47) and HC (0.054 ± 2.33). The mean ADII was above zero in all groups, indicating a slightly pro-inflammatory diet. The differences in scores for vitamins and minerals varied per micronutrient. Further details are given in Appendix A, Table A3.

**Figure A1:**
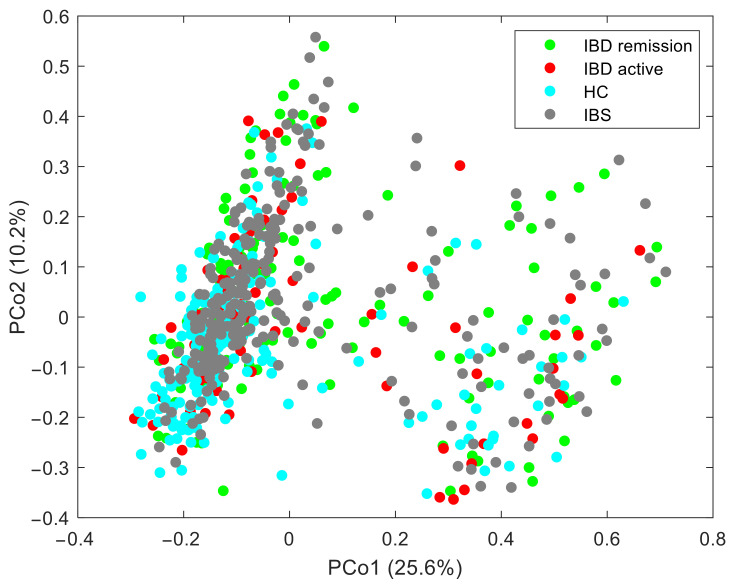
Principle coordinate analysis (PCoA) score plot based on food products. IBD = inflammatory bowel disease; IBS = irritable bowel syndrome; HC = healthy controls; PCo = principle coordinate.

**Figure A2:**
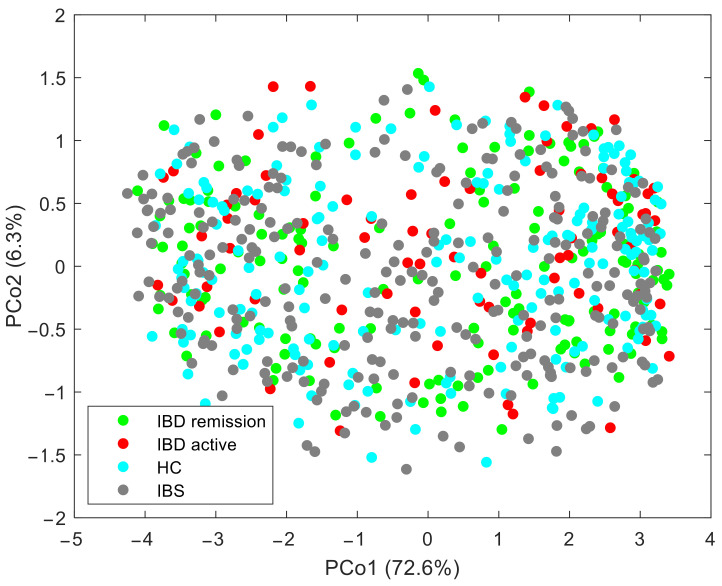
Principle coordinate analysis (PCoA) score plot based on nutrients—PCo1 and PCo2. IBD = inflammatory bowel disease; IBS = irritable bowel syndrome; HC = healthy controls; PCo = principle coordinate.

**Figure A3:**
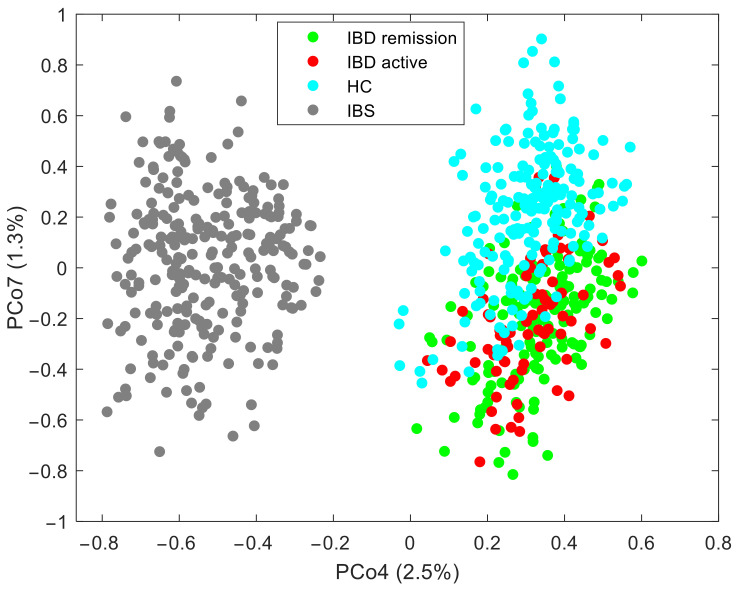
Principle coordinate analysis (PCoA) score plot based on nutrients—PCo4 and PCo7. IBD = inflammatory bowel disease; IBS = irritable bowel syndrome; HC = healthy controls; PCo = principle coordinate. The most important components that cause the distinction between IBS and the rest are visible along PCo4. It is characterised by, among others, a combination of a lower intake of zinc, caloric intake, selenium, vitamin B12, magnesium and iron in IBS compared to IBD and HC.

The explorative URF resulted in principal coordinate analysis (PCoA) score plots, which showed no relevant grouping based on either food items or nutrients (Appendix B, Figure A1 and Figure A2) when considering PCo1 and PCo2. Only PCo4 and PCo7 of nutrient intake data (Figure A3) showed a separation of IBS as compared to IBD and HC, explaining only 3.8% of the total variance. More details are given in Appendix B.

### 3.3. Disease Phenotypes

Separate explorative analyses on disease phenotypes showed that the DHD-2015 was significantly lower in active as compared to remissive IBD patients (64.77 ± 15.38 vs. 71.15 ± 16.72, *p* = 0.004), and also in CD compared to UC (65.47 ± 15.94 vs. 75.71 ± 15.61, *p* < 0.001). No significant differences were found for the DHD-2015 between IBS subtypes, nor did the ADII differ between disease phenotypes. Further details are given in the Supplementary Materials Tables S1–S3.

### 3.4. Intestinal Inflammation

Mean faecal calprotectin levels (Table 2) were significantly higher in IBD patients (197.3 ± 426.3 μg/g) as compared to IBS (64.6 ± 87.1 μg/g, *p* = 0.001) and HC (39.3 ± 63.6 μg/g, *p* < 0.001), but no differences were found between IBS and HC (*p* > 0.999).

Based on the multivariable linear regression analysis (Table 3), the DHD-2015 was associated with faecal calprotectin in IBD patients (b = −4.009, *p* = 0.006), but not in IBS patients or HC (IBS: *p* = 0.991; HC: *p* = 0.144). Faecal calprotectin levels were not associated with the ADII in either of the groups (IBD: *p* = 0.229; IBS: *p* = 0.474; HC: *p* = 0.267).

### 3.5. GI Symptoms

IBS patients scored significantly higher on all GSRS subdomains as compared to IBD and HC individuals (*p* < 0.001 for all comparisons, Table 2). In addition, IBD patients scored significantly higher than HC on subdomains abdominal pain (*p* = 0.002), diarrhoea syndrome (*p* < 0.001) and indigestion syndrome (*p* < 0.001), but not for other subdomains.

Using a multivariable linear regression analysis (Table 3), abdominal pain was significantly associated with the ADII in IBD patients (b = 0.194, *p* = 0.004), and with the DHD-2015 in IBS patients (b = −0.012, *p* = 0.023). Furthermore, in IBS patients, reflux syndrome was significantly associated with the DHD-2015 (b = −0.016, *p* = 0.004). No significant associations were found for the GSRS subdomains constipation syndrome, diarrhoea syndrome and indigestion syndrome. In HC, none of the associations were significant.

## 4. Discussion

We found that diet quality was significantly lower in IBD and IBS patients as compared to HC. However, there was no difference in the dietary inflammatory potential between groups based on the ADII. Furthermore, our results showed that a lower diet quality was associated with more intestinal inflammation in IBD, while it was associated with higher symptom scores in IBS patients. A more pro-inflammatory diet was only associated with higher abdominal pain scores in IBD patients.

Overall diet quality was lower in both IBD and IBS patients compared to HC, being especially lower for dairy and high-fibre foods such as wholegrain products, fruit and vegetables and legumes. This is in line with previous studies reporting these food groups as perceived food culprits in both patient groups [9,10], and with studies indicating that IBD and IBS patients are at increased risk for nutritional deficiencies and malnutrition [37,38,39]. This emphasises the importance of good dietary advice when avoiding certain food products.

Whereas overall diet composition cannot be used to differentiate between IBD and IBS, it should be noted that some differences can be found, such as the lower intake of wholegrain products and red meat in IBS. Additionally, it is important to note that the DHD-2015 was validated in healthy subjects, while IBD and IBS patients may need other recommendations. For example, IBD patients with active disease have been reported to require a higher protein intake than those in remission or healthy individuals [40]. Further, patients may need higher intakes due to more loss (diarrhoea) and less absorption of nutrients [37,38]. This further stresses the relevance of adequate dietary advice, using a tailored approach and taking into account disease characteristics and nutritional status.

Diet in general, and specific food items in particular, can impact mechanisms that may contribute to disease course in IBD and IBS directly by impacting host immune function or indirectly via the intestinal microbiome and barrier disruptive effects [7,29]. We therefore evaluated the ADII as an indicator for the inflammatory potential of the overall diet, and found a wide range with on average a slightly pro-inflammatory index (i.e., above 0) in all groups, which did, however, not differ between the groups. In future studies, it would be interesting to further investigate whether this could impact intestinal health differently in susceptible patients as compared to healthy control subjects. Additionally, the ADII takes into account that foods are generally not consumed in isolation, but may miss over- or underconsumption of specific nutrients. In line with this, the standardised energy-corrected intake of nutrients used for this score is important to avoid overestimation of the effect of certain nutrients; however, this may also partially explain why we found no differences between groups, despite some differences in the absolute intake of several pro- and anti-inflammatory components. A limited group difference was also illustrated by our explorative URF analyses, which, based on PCo4 and PCo7 (explaining < 4% of variance), indicated a minor but clear distinction between the nutrient intake of IBS patients compared to IBD and HC (see Appendix B). The URF was added to identify any relevant unknown dietary patterns, but findings should be interpreted with care as no distinction was found by PCo1 and PCo2. This further illustrates the complexity of interpreting dietary data, and the need for longitudinal studies on the exact role of both dietary patterns and specific nutrients and product groups in the development of intestinal inflammation and symptoms, studied separately for these patient groups because of potential differences.

In line with our results, a previous study using the Dietary Inflammatory Index (DII) in IBD patients also pointed towards a slightly pro-inflammatory diet [41]. The (A)DII was not previously assessed in IBS, but a previous study using the EDII found a pro-inflammatory diet being associated with higher odds of having IBS [42]. The EDII [14] is based on food groups rather than nutrients. We chose not to incorporate the EDII in our analyses because the defined food groups were not representative for the Dutch dietary intake.

In our study, no association was found between the ADII and faecal calprotectin as a marker for intestinal inflammation in IBD nor in IBS. In addition, no difference was observed in the ADII score between remissive versus active IBD. These findings are in line with a study by Mirmiran et al. that found no association between the inflammatory potential of diet and disease severity, as defined by the CDAI and Mayo score [43]. In contrast, Lamers et al. found that the DII was significantly lower in IBD patients in remission, compared to IBD patients with mild or moderate active disease, and that a more pro-inflammatory diet was associated with higher Clinical Disease Activity Index (sCDAI) in CD patients [41]. It should, however, be considered that clinical activity indices do not necessarily correlate with active inflammation [41].

Although a more pro-inflammatory diet did not correlate significantly with low diet quality in either of our groups, a lower diet quality was significantly associated with more intestinal inflammation in IBD, but not in IBS. Diet quality as scored by the DHD-2015 was also significantly lower in active IBD patients compared to IBD patients in remission. We cannot exclude that the observation (in part) was due to related symptoms, but we do not have sufficient power to draw firm conclusions on this. In addition, it is important to note the limitation of the cross-sectional design and that the relation between diet quality and intestinal inflammation could be bidirectional. A low intake of favourable nutrients, such as antioxidants and fibres—the latter of which leads to enhanced production of short-chain fatty acids—can increase the risk of a flare [44]. On the other hand, patients with active disease (i.e., more inflammation) often change their diet in an attempt to mitigate symptom burden, which can result in poorer diet quality [45]. Thus, longitudinal studies are necessary to gain more insight in the causality of such associations.

As diet can also play a role in symptom onset via, for example, osmotic effects and distension, we investigated the association with symptom domains associated with IBS that are also common in IBD. We found a more pro-inflammatory diet, but not an overall diet quality to be associated with more abdominal pain in IBD patients. Although abdominal pain scores were not different in active versus quiescent IBD patients, diarrhoea was more common.

Based on our results, the inflammatory potential of the diet does not seem to be the driving factor for symptom severity in IBS, which is in line with a previous study [42]. However, in IBS, a lower diet quality was associated with more GI symptoms. Again, these associations could be bidirectional. Multiple previous studies reported both IBD and IBS patients adjusting their diet because of food-related symptoms, resulting in a less healthy diet [10,46,47,48,49,50,51]. Although data on individual dietary advice were not available for the current study, a recent national Dutch survey showed that 71% of IBS patients indicated having changed their diet because of symptoms, of which only 30% were supervised by a dietitian [52]. Notwithstanding, in the current study, symptom scores were still increased as compared to controls and a lower diet quality can also (further) exacerbate symptoms. This again stresses the importance of further investigating the causality of such associations using longitudinal studies. Hereby, it would be interesting to add further markers for malnutrition and potential underlying mechanisms related to, e.g., the immune system and the microbiome.

A strength of our study was the assessment of the overall dietary patterns in different patient populations and HC, rather than just single foods or nutrients in homogeneous study populations. A limitation was that the FFQ was not validated for the calculation of micronutrients intake, and that use of nutritional supplements was not incorporated into the analysis. Furthermore, some anti-inflammatory components, such as caffeine, quercetin and garlic, could not be calculated. Therefore, the ADII might slightly overestimate the pro-inflammatory potential of the diet.

## 5. Conclusions

In this study, we investigated the relationship of the adherence to the Dutch dietary guidelines (using the DHD-2015) and the inflammatory potential of the diet (using the ADII) with inflammatory markers and GI symptoms in both IBD and IBS patients that share culprit foods.

A low overall diet quality and a slightly pro-inflammatory diet was observed in both IBD and IBS patients, indicating the need of improving diet quality with adequate nutritional guidance. Furthermore, diet quality was associated with faecal calprotectin in IBD and with several GI symptoms in IBS, whereas the inflammatory potential of the diet was only associated with GI symptoms in IBD. These differences between the studied patient groups may point to differential roles in the pathophysiology. However, due to the cross-sectional design, we cannot draw firm conclusions on the direction or presence of causality between diet, intestinal inflammation and GI symptoms. Our findings support the need for longitudinal studies to further investigate the role of dietary factors in the development of flares and predominant symptoms.

## Figures and Tables

**Table 1:** Baseline characteristics in inflammatory bowel disease (IBD) patients, irritable bowel syndrome (IBS) patients and healthy controls (HC).

Characteristics	IBD Patients (*n* = 238)	IBS Patients (*n* = 261)	HC (*n* = 195)	*p*-Value
Age (years)	45.7 ± 14.8	43.3 ± 17.0	44.4 ± 18.9	0.285
Sex				<0.001
Male	47.1%	25.3%	36.9%	
Female	52.9%	74.7%	63.1%	
BMI (kg/m^2^) *	25.5 ± 4.2	25.0 ± 4.6	23.9 ± 3.8	<0.001
Smoking **				<0.001
Active smoker	20.4%	23.6%	6.7%	
Former smoker	41.7%	24.4%	31.8%	
Never smoker	37.9%	52.0%	61.5%	
IBD phenotype				
Crohn’s disease	65.5%	n/a	n/a	n/a
Ulcerative colitis	34.5%	n/a	n/a	n/a
Age of onset **				
A1—below 17 years old	5.9%	n/a	n/a	n/a
A2—17–40 years old	64.0%	n/a	n/a	n/a
A3—above 40 years old	30.1%	n/a	n/a	n/a
Behaviour of Crohn’s disease at inclusion				
B1—non-stricturing, non-penetrating	57.1%	n/a	n/a	n/a
B2—stricturing	17.9%	n/a	n/a	n/a
B3—penetrating	25.0%	n/a	n/a	n/a
Location of Crohn’s disease at inclusion				
L1—ileal	23.7%	n/a	n/a	n/a
L2—colonic	16.7%	n/a	n/a	n/a
L3—ileocolonic	59.6%	n/a	n/a	n/a
L4—upper-GI modifier	10.3%	n/a	n/a	n/a
Extent of ulcerative colitis (UC) at inclusion **				
E1—ulcerative proctitis	11.1%	n/a	n/a	n/a
E2—left sided UC (distal UC)	39.5%	n/a	n/a	n/a
E3—extensive UC (pancolitis)	49.4%	n/a	n/a	n/a
Disease activity at inclusion				
Active disease	34.9%	n/a	n/a	n/a
Remission	61.5%	n/a	n/a	n/a
Time to last flare (months)	37.7 ± 67.7	n/a	n/a	n/a
Bowel resection at inclusion				
Yes	23.1%	n/a	n/a	n/a
No	76.9%	n/a	n/a	n/a
Symptom score *				
Harvey Bradshaw Index	2.9 ± 3.4	n/a	n/a	n/a
Simple Clinical Colitis Activity Index	1.2 ± 1.8	n/a	n/a	n/a
IBS Subtype				
Constipation predominant IBS	n/a	21.5%	n/a	n/a
Diarrhoea predominant IBS	n/a	35.6%	n/a	n/a
Mixed stool pattern IBS	n/a	39.5%	n/a	n/a
Unspecified subtype IBS	n/a	3.4%	n/a	n/a
Disease duration (years) **	11.5 ± 10.1	14.4 ± 14.9	n/a	n/a
Medication ***				
No medication	14.3%	26.8%	52.8%	<0.001
5-ASA, local immunosuppressants, or local corticosteroids	17.6%	n/a	n/a	n/a
Systemic corticosteroids	0.4%	n/a	n/a	n/a
Immunomodulators	22.7%	n/a	n/a	n/a
Biologicals	45.0%	n/a	n/a	n/a
PPIs	n/a	20.7%	3.1%	<0.001
NSAIDs	n/a	24.9%	20.0%	0.217
Laxatives	n/a	18.4%	0.0%	n/a
Spasmolytic drugs	n/a	14.2%	0.0%	n/a
Antihypertensive drugs	n/a	15.3%	13.3%	0.550
Statins	n/a	10.0%	7.7%	0.402
Antidepressant drugs	n/a	10.0%	3.6%	0.009

IBD = inflammatory bowel disease; IBS = irritable bowel syndrome; HC = healthy controls; BMI = Body Mass Index; 5-ASA = 5-aminosalicylic acid; PPIs = proton pump inhibitors; NSAIDs = non-steroidal anti-inflammatory drugs; n/a = not applicable or not available. * Missing data from max. 25 participants per subgroup. ** Missing data from max. 3 participants per subgroup. *** Missing data from 4 IBS patients. Medication for IBD patients was classified as the highest category of use. For IBS medication, only the most important medications are displayed. Other medication included prokinetics, anti-diarrhoeal drugs, oral contraceptives, antipsychotic drugs and antibiotics. Continuous data expressed as mean ± standard deviation (SD). Categorical data expressed as percentages of total group (IBD, IBS or HC). The differences between IBD, IBS and HC were tested with ANOVA and post-hoc Bonferroni correction for continuous data, and the Chi-square test with Fisher for categorical data.

**Table 2:** Baseline characteristics in inflammatory bowel disease (IBD) patients, irritable bowel syndrome (IBS) patients and healthy controls (HC).

Disease Characteristics	IBD Patients (*n* = 238)		IBS Patients (*n* = 261)		HC (*n* = 195)		*p*-Value
Calprotectin (μg/g)	197.3 ± 426.3	(*n* = 209)	64.4 ± 87.1	(*n* = 90)	39.3 ± 63.6	(*n* = 148)	<0.001
GSRS							
Abdominal pain	2.1 ± 1.0	(*n* = 80)	3.3 ± 1.2	(*n* = 258)	1.6 ± 0.7	(*n* = 194)	<0.001
Constipation syndrome	1.9 ± 1.1	(*n* = 70)	3.4 ± 1.3	(*n* = 257)	1.6 ± 0.8	(*n* = 193)	<0.001
Diarrhoea syndrome	2.7 ± 1.5	(*n* = 77)	3.3 ± 1.5	(*n* = 258)	1.4 ± 0.6	(*n* = 194)	<0.001
Indigestion syndrome	2.7 ± 1.2	(*n* = 80)	4.1 ± 1.3	(*n* = 256)	2.0 ± 0.8	(*n* = 193)	<0.001
Reflux syndrome	1.4 ± 0.8	(*n* = 80)	2.2 ± 1.4	(*n* = 258)	1.2 ± 0.5	(*n* = 195)	<0.001

IBD = inflammatory bowel disease; IBS = irritable bowel syndrome; HC = healthy controls; GSRS = Gastrointestinal Symptom Rating Scale. Continuous data expressed as mean ± standard deviation (SD). The differences between IBD, IBS and HC were tested with ANOVA and post-hoc Bonferroni correction.

**Table 3:** Results of multivariable linear regression analysis (after adjustment for possible confounders) of dietary indices for disease parameters.

	IBD Patients			IBS Patients			HC		
Disease Characteristics	β	95% CI	*p*-Value	β	95% CI	*p*-Value	β	95% CI	*p*-Value
Faecal calprotectin									
DHD-2015	−4.009	−6.875; −1.143	0.006	0.006	−1.105; 1.117	0.991	−0.506	−1.186; 0.175	0.144
ADII	11.259	−7.157; 29.675	0.229	−2.880	−10.853; 5.093	0.474	3.036	−2.349; 8.421	0.267
Abdominal pain									
DHD-2015	−0.006	−0.024; 0.011	0.460	−0.012	−0.022; −0.002	0.023	−0.001	−0.006; 0.005	0.784
ADII	0.194	0.065; 0.323	0.004	0.005	−0.065; 0.074	0.895	0.014	−0.028; 0.056	0.510
Constipation syndrome									
DHD-2015	−0.007	−0.025; 0.011	0.454	0.008	−0.001; 0.017	0.075	0.001	−0.006; 0.008	0.724
ADII	−0.015	−0.161; 0.132	0.843	−0.027	−0.090; 0.036	0.402	−0.030	−0.081; 0.020	0.235
Diarrhoea syndrome									
DHD-2015	−0.017	−0.042; 0.008	0.168	0.000	−0.011; 0.011	0.989	0.000	−0.005; 0.005	0.997
ADII	0.173	−0.021; 0.367	0.079	0.023	−0.052; 0.097	0.545	−0.019	−0.060; 0.022	0.358
Indigestion syndrome									
DHD-2015	−0.016	−0.035; 0.003	0.101	−0.001	−0.012; 0.011	0.906	0.001	−0.006; 0.009	0.740
ADII	0.107	−0.049; 0.262	0.174	−0.007	−0.083; 0.070	0.857	−0.030	−0.086; 0.026	0.291
Reflux syndrome									
DHD-2015	−0.000	−0.014; 0.013	0.970	−0.016	−0.027; −0.005	0.004	0.002	−0.003; 0.007	0.395
ADII	−0.064	−0.173; 0.044	0.240	0.058	−0.018; 0.134	0.133	−0.014	−0.050; 0.022	0.430

IBD = inflammatory bowel disease; IBS = irritable bowel syndrome; HC = healthy controls; β = regression coefficient; 95% CI = 95% confidence interval; DHD-2015 = Dutch healthy diet index 2015; ADII = Adapted Dietary Inflammatory Index. Faecal calprotectin was measured in μg/g (marker for intestinal inflammation). Abdominal pain, constipation syndrome, diarrhoea syndrome, indigestion syndrome and reflux syndrome were defined using the Gastrointestinal Symptom Rating Scale. Analyses were performed using multivariable linear regression, and were corrected for: age, sex, smoking, BMI, disease specific medication (all subgroups), plus phenotype, disease duration (years) and age of onset according to the Montreal classification for IBD, or plus subtype for IBS.

## Data Availability

The data presented in this study are available on request from the corresponding author.

## Supplementary Materials

The following supporting information can be downloaded at: https://www.mdpi.com/article/10.3390/nu14091945/s1, Table S1: Dietary indices, intestinal inflammation and gastrointestinal symptoms per disease activity status for inflammatory bowel disease; Table S2: Dietary indices, intestinal inflammation and gastrointestinal symptoms per inflammatory bowel disease phenotype; Table S3: Intestinal inflammation and gastrointestinal symptoms per irritable bowel syndrome subtype.

## Appendix A

Detailed information on the calculation of the dietary indices, i.e., the DHD-2015 and the ADII, can be found in Appendix A. Table A1: Categorisation of food items derived from the Food Frequency Questionnaire (FFQ) into the Dutch Healthy Diet Index 2015 (DHD-2015); Table A2: Absolute intake and Dutch Healthy Diet Index (DHD-2015) score per component; Table A3: Absolute intake per component of the Adapted Dietary Inflammatory Index (ADII).

**Table A1:** Categorisation of food items derived from the Food Frequency Questionnaire (FFQ) into the Dutch Healthy Diet Index 2015 (DHD-2015).

Component DHD-2015	Included FFQ Food Items	
	Dutch Item Name	English Item Name
1. Vegetables	Gekookte bloemkool en broccoli	Boiled cauliflower or broccoli
	Gekookte koolsoorten (witte-, rode-, spits-, groene-, savooie-, Chinese-, boeren-en zuurkool)	Boiled cabbage varieties (white-, red-, oxheart-, green-, savoy-, Chinese cabbage, kale, sauerkraut)
	Gekookte ui en prei	Boiled onion and leak
	Overige gekookte groente	Other boiled vegetables
	Rauwe groente	Raw vegetables
2. Fruit	Appels (vers)	Apples (fresh)
	Banaan (vers)	Bananas (fresh)
	Citrusfruit (vers)	Citrus fruits (fresh)
	Overig vers fruit	Other fruits (fresh)
3a. Wholegrain products	All bran	All bran cereal
	Bruin brood	Brown bread
	Meergranen brood	Multigrain bread
	Papgranen (Brinta, havermout, enz.)	Porridge grains (Brinta, oatmeal, etc.)
	Roggebrood	Rye bread
	Volkoren brood	Whole grain bread
3b. Refined grain products	Beschuit, knackebrod en crackers	Plain rusk, Swedish crispbread and crackers
	Cornflakes	Cornflakes
	Croissants	Croissants
	Muesli, cruesli	Muesli and granola
	Overige ontbijtproducten	Other breakfast cereals, breads, etc.
	Pasta	Pasta
	Rijst	Rice
	Rozijnen-, krenten- of mueslibrood	Raisin bread, plum loaf, muesli bread
	Wit brood	Plain white bread
4. Legumes	Peulvruchten	Legumes
5. Nuts	Noten, notenmix, studenten-haver	Nuts, nut mixes, trail mix
6a. Dairy	Crème fraîche en andere bereidingsroom	Crème fraiche and other cooking creams
	Halfvolle melk	Reduced-fat milk
	Halfvolle (vruchten)yoghurt	Reduced-fat (fruit) yoghurt
	Karnemelk	Buttermilk
	Koffiemelk en -creamer	Coffee milk and coffee creamer
	Kwark en vruchtenkwark	Quark or curd
	Magere (vruchten)yoghurt	Low-fat (fruit) yoghurt
	Magere melk	Low-fat milk
	Pappen	Porridge
	Roomijs en ijs(jes) op melkbasis	Milk-based ice-cream
	Slagroom en topping	Whipped cream and toppings
	Vla en pudding	Custard and pudding
	Volle melk	Whole milk
	Volle (vruchten)yoghurt	Whole yoghurt
6b. Cheese	Kaas	Cheese
	Roomkaas en buitenlandse kaas	Cream cheese or foreign cheese
	Smeerkaas en zuivelspread	Cheese spread or dairy spread
7a. Fish—Oily	Forel, tonijn (vers, diepvries, in blik)	Trout or tuna (fresh, frozen or canned)
	Gerookte en gestoomde vis (bv. zalm, makreel, bokking)	Smoked or steamed fish (salmon, mackerel, herring, etc.)
	Haring en sardines	Herring and sardines
	Zalm, makreel, paling, pan-haring, enz. (vers, diepvries, in blik)	Fatty fish (salmon, mackerel, eel, etc.)
7b. Fish—Lean	Kabeljauw, schol, schelvis, kool-vis, tong, enz.	Low-fat white fish (cod, plaice, haddock, pollock, sole, etc.)
	Lekkerbekje of kibbeling	Fried fillet of haddock
	Schaal- en schelpdieren	Crustaceans and shellfish
	Vissticks	Fish fingers
8. Tea	Thee	Tea
9a. Solid cooking fats	Bak en braadproduct (vast)	Solid baking and roasting product
	Frituurvet (vast)	Solid frying product
	Halfvolle roomboter	Reduced-fat butter
	Margarine in pakje	Margarine (foil)
	Roomboter	Butter
	Spekvet of rundervet	Bacon fat or beef fat
9b. Liquid cooking fats	Dieethalvarine	Diet low-fat margarine
	Dieetmargarine	Diet margarine
	Halvarine	Low-fat margarine
	Halvarine met plantensterolen/stanolen	Low-fat margarine with plant sterols/stanols
	Laagvet halvarine product	Low-fat margarine product
	Margarine in kuipje	Margarine (tub)
	Margarine met plantensterolen/stanolen	Margarine with plant sterols/stanols
	Olijfolie	Olive oil
	Vloeibaar bak en braadproduct	Liquid baking and roasting product
	Vloeibaar frituurproduct	Liquid frying product
	Vloeibare margarine	Liquid margarine
	Zonnebloemolie, sojaolie, slaolie, enz. (geen olijfolie)	Sunflower oil, salad oil, etc.
10. Coffee	No data available on filtered vs. unfiltered	
11. Red meat	Gehakt	Minced meat
	Lamsvlees of schapenvlees	Lamb, hogget, mutton
	Orgaanvlees	Organ meats, giblets
	Overig varkensvlees	Other types of pork meat
	Overige soorten vlees en wild	Other types of game meat
	Runderbiefstuk, rundertartaar, runder-baklap, runderbraadlap, runderrosbief	Beef steak, roast, casserole, tartare, etc.
	Runderentrecote, runder-braadworst, rundersukadelap, runderriblap, doorregen runder-lap	Beef entrecote, bratwurst, sirloin steak, etc.
	Varkenshaas, varkensschnitzel, varkensfricandeau, varkens-hamlap	Pork tenderloin, cutlet, fillet, ham steak, etc.
	Varkenskarbonade (schouder-, rib- en haas karbonade)	Pork chops (shoulder, rib or fillet chops)
12. Processed meat	(Smeer)leverworst, paté, leverpastei, leverkaas, berliner	Liverwurst spread, paté, liver pate, liver cheese, Berliner liver sausage
	Boterhamworst, gekookte worst, palingworst, gebraden gehakt	Cold cut sausages (pork)
	Cervelaatworst, snijworst, metworst, salami	Cold cut sausages (beef)
	Gekookte lever	Cooked liver
	Ham	Ham
	Hamburger	Hamburger
	Overige soorten vleeswaar	Other types of cold cuts
	Rookvlees, fricandeau, rosbief, casselerrib, kipfilet, kiprollade	Cold cuts varieties (including poultry)
	Rookworst of knakworst	Smoked sausage, frankfurters
	Speklappen en spekjes	Bacon, pork belly
	Varkensbraadworst en slavink	Pork sausages
13. Sweetened beverages and fruit juices	(Light) vruchtendrank (met zoetstof), dubbeldrank, multi-vruchtendrank	Light fruit drinks
	Chocolademelk	Chocolate milk
	Drinkontbijt	Breakfast drink
	Drinkyoghurt en andere zuivel-dranken	Sweetened dairy drinks
	Frisdrank, vruchtenlimonade, sportdrank en energiedrank	Soda, lemonade, sport drinks, energy drinks
	Milkshake	Milkshake
	Vruchtensap uit pak of fles of versgeperst	Fruit juice (fresh or bottle)
14. Alcohol	Alcohol (nutrient), met behulp van producten:	Alcohol (nutrient), assessed using products:
	Bier	Beer
	Breezer	Breezer
	Sherry, port, vermout, enz.	Fortified wines: Sherry, Port, Vermouth, etc.
	Sterke drank	Spirits
	Wijn	Wine
15. Salt	No data available	

DHD-2015 = Dutch Healthy Diet Index 2015; FFQ = Food Frequency Questionnaire. More details on the components used to calculate the DHD-2015 can be found in Looman et al. [12].

**Table A2:** Absolute intake and Dutch Healthy Diet Index (DHD-2015) score per component.

	IBD Patients (*n* = 238)		IBS Patients (*n* = 261)		HC (*n* = 195)		
	Mean ± SD	Range	Mean ± SD	Range	Mean ± SD	Range	*p*-Value
Absolute intake							
1. Vegetables (g/day)	108.52 ± 68.50	0.00–344.95	118.64 ± 87.91	0.00–705.74	133.72 ± 103.42	7.57–888.62	0.011
2. Fruit (g/day)	134.42 ± 113.59	0.00–579.35	148.13 ± 108.91	0.00–495.42	185.20 ± 143.85	0.00–882.64	<0.001
3a. Wholegrain products (g/day)	113.36 ± 73.58	0.00–431.56	95.83 ± 69.42	0.00–420.00	117.89 ± 71.34	0.00–334.00	0.002
3b. Refined grain products (g/day)	90.89 ± 56.87	0.00–361.15	85.52 ± 60.89	1.60–341.67	103.98 ± 77.77	0.00–534.50	0.010
3c. Wholegrain/Refined grain ratio	3.31 ± 10.20	0.00–115.28	2.44 ± 9.15	0.00–138.91	2.07 ± 3.38	0.00–33.39	0.278
4. Legumes (g/day)	17.30 ± 36.96	0.00–305.08	14.71 ± 24.13	0.00–209.90	20.22 ± 28.65	0.00–173.29	0.158
5. Nuts (g/day)	5.27 ± 12.81	0.00–154.27	5.95 ± 11.99	0.00–92.69	7.17 ± 12.96	0.00–121.58	0.291
6a. Milk and yoghurt (g/day)	189.95 ± 173.10	0.00–1344.04	191.65 ± 169.38	0.00–1243.13	232.28 ± 195.19	0.00–1432.13	0.023
6b. Cheese (g/day)	25.88 ± 23.01	0.00–129.14	24.15 ± 23.08	0.00–120.42	31.02 ± 30.16	0.00–164.72	0.014
6c. Dairy (g/day)	211.49 ± 174.18	0.00–1344.64	211.48 ± 171.75	0.00–1283.13	255.60 ± 194.37	0.00–1438.58	0.015
7a. Fish—Oily (g/day)	8.79 ± 11.54	0.00–69.32	10.65 ± 13.82	0.00–94.44	11.11 ± 12.02	0.00–73.51	0.114
7b. Fish—Lean (g/day)	12.59 ± 15.15	0.00–113.86	10.65 ± 12.88	0.00–114.34	10.34 ± 10.39	0.00–51.78	0.136
7c. Fish total (g/day)	11.80 ± 12.05	0.00–73.32	13.59 ± 14.29	0.00–98.44	14.07 ± 12.50	0.00–77.51	0.151
8. Tea (g/day)	248.06 ± 318.31	0.00–1625.00	270.67 ± 319.29	0.00–1950.00	260.62 ± 319.02	0.00–1950.00	0.731
9a. Solid cooking fats (g/day)	3.94 ± 5.99	0.00–37.77	3.98 ± 7.18	0.00–61.93	3.83 ± 6.96	0.00–48.71	0.971
9b. Liquid cooking fats (g/day)	26.14 ± 15.10	0.00–89.31	21.92 ± 13.05	0.00–75.26	25.91 ± 15.03	0.00–67.44	0.001
9c. Solid/liquid cooking fats ratio	48.60 ± 133.27	0.00–1144.50	35.69 ± 98.58	0.00–840.50	62.05 ± 295.26	0.00–3123.50	0.446
10a. Coffee, unfiltered	Unknown		Unknown		Unknown		
10b. Coffee, filtered	Unknown		Unknown		Unknown		
11. Red meat (g/day)	55.72 ± 36.14	0.00–183.73	47.93 ± 30.26	0.00–139.86	46.11 ± 38.50	0.00–220.16	0.008
12. Processed meat (g/day)	44.34 ± 34.46	0.00–208.84	37.95 ± 35.06	0.00–265.38	29.12 ± 26.58	0.00–141.43	<0.001
13. Sweetened beverages and fruit juices (g/day)	188.10 ± 235.71	0.00–1528.62	195.43 ± 235.17	0.00–1535.54	166.02 ± 213.45	0.00–1569.09	0.384
14. Alcohol (g/day)	8.73 ± 11.68	0.00–72.76	8.30 ± 14.94	0.00–147.87	11.59 ± 12.86	0.00–95.08	0.022
15. Salt (g/day)	Unknown		Unknown		Unknown		
DHD-2015 score per component							
1. Vegetables	5.20 ± 2.94	0.00–10.00	5.38 ± 3.03	0.00–10.00	5.90 ± 2.96	0.38–10.00	0.043
2. Fruit	5.54 ± 3.74	0.00–10.00	6.19 ± 3.64	0.00–10.00	6.88 ± 3.47	0.00–10.00	0.001
3a. Wholegrain products	3.96 ± 1.53	0.00–5.00	3.62 ± 1.75	0.00–5.00	4.10 ± 1.49	0.00–5.00	0.004
3b. Refined grain products	0.74 ± 1.24	0.00–5.00	0.58 ± 0.93	0.00–5.00	0.64 ± 1.02	0.00–5.00	0.236
3c. Wholegrain/Refined grain ratio	4.71 ± 2.28	0.00–10.00	4.21 ± 2.28	0.00–10.00	4.74 ± 2.07	0.00–10.00	0.013
4. Legumes	5.40 ± 4.57	0.00–10.00	5.39 ± 4.61	0.00–10.00	6.33 ± 4.52	0.00–10.00	0.056
5. Nuts	2.42 ± 3.46	0.00–10.00	2.63 ± 3.43	0.00–10.00	3.39 ± 3.59	0.00–10.00	0.011
6. Dairy	5.48 ± 3.11	0.00–10.00	5.52 ± 3.11	0.00–10.00	6.10 ± 3.06	0.00–10.00	0.076
7. Fish	5.80 ± 3.64	0.00–10.00	6.25 ± 3.51	0.00–10.00	6.72 ± 3.56	0.00–10.00	0.029
8. Tea	4.04 ± 3.91	0.00–10.00	4.46 ± 3.92	0.00–10.00	4.29 ± 3.82	0.00–10.00	0.490
9. Fats and oils	6.72 ± 3.89	0.00–10.00	6.52 ± 3.97	0.00–10.00	7.24 ± 3.87	0.00–10.00	0.141
10. Coffee	Unknown		Unknown		Unknown		
11. Red meat	7.09 ± 3.58	0.00–10.00	7.61 ± 3.14	0.00–10.00	7.92 ± 3.34	0.00–10.00	0.032
12. Processed meat	3.36 ± 3.43	0.00–10.00	4.11 ± 3.58	0.00–10.00	5.04 ± 3.51	0.00–10.00	<0.001
13. Sweetened beverages and fruit juices	5.01 ± 3.89	0.00–10.00	4.87 ± 3.84	0.00–10.00	5.33 ± 3.81	0.00–10.00	0.442
14. Alcohol	8.23 ± 3.31	0.00–10.00	8.47 ± 3.25	0.00–10.00	7.47 ± 3.75	0.00–10.00	0.006
15. Salt	Unknown		Unknown		Unknown		
DHD-2015	68.998 ± 16.53	24.64–115.58	71.608 ± 16.58	21.57–111.34	77.347 ± 17.43	32.47–119.10	<0.001

IBD = inflammatory bowel disease; IBS = irritable bowel syndrome; HC = healthy controls; DHD-2015 = Dutch Healthy Diet Index 2015. Continuous data expressed as mean ± standard deviation (SD). Differences between IBD, IBS and HC were tested exploratively with ANOVA and post-hoc Bonferroni correction, without correction for multiple testing. More details on the calculation of the DHD-2015 components can be found in Looman et al. [12].

**Table A3:** Absolute intake per component of the Adapted Dietary Inflammatory Index (ADII).

	IBD Patients (*n* = 238)		IBS Patients (*n* = 261)		HC (*n* = 195)		
	Mean ± SD	Range	Mean ± SD	Range	Mean ± SD	Range	*p*-Value
Energy (kcal/day)	2179.95 ± 634.33	878.90–3962.92	1939.59 ± 603.87	642.72–3834.86	2180.41 ± 622.89	821.61–3868.48	<0.001
Protein (g/day)	78.69 ± 23.58	29.87–169.96	72.33 ± 21.24	19.36–148.76	79.18 ± 23.87	29.17–153.79	0.001
Saturated fatty acids (g/day)	31.40 ± 11.52	7.15–66.65	27.59 ± 10.62	5.64–70.69	30.00 ± 10.52	9.06–61.22	<0.001
Mono-unsaturated fatty acids (g/day)	31.78 ± 12.19	8.89–75.79	28.05 ± 10.86	5.80–68.61	30.41 ± 10.80	9.23–80.93	0.001
Trans fatty acids (g/day)	1.33 ± 0.53	0.29–3.01	1.21 ± 0.53	0.17–3.64	1.33 ± 0.51	0.35–2.64	0.014
n-3 poly-unsaturated fatty acids (g/day)	2.29 ± 0.85	0.58–5.38	2.09 ± 0.80	0.38–5.34	2.34 ± 0.92	0.83–5.07	0.003
n-6 poly-unsaturated fatty acids (g/day)	15.87 ± 6.58	3.78–49.71	13.68 ± 6.30	2.62–51.47	15.44 ± 6.81	4.91–52.63	<0.001
Cholesterol (mg/day)	207.22 ± 86.19	46.51–631.17	195.11 ± 86.47	8.34–685.68	198.52 ± 82.95	34.40–628.82	0.272
Carbohydrate (g/day)	237.02 ± 73.11	77.75–477.29	208.81 ± 71.70	45.49–464.17	237.22 ± 72.12	84.69–471.34	<0.001
Fibre (g/day)	22.33 ± 8.06	4.81–56.04	20.52 ± 7.13	2.17–45.53	24.67 ± 8.47	9.10–55.26	<0.001
Ethanol (g/day)	8.73 ± 11.68	0.00–72.76	8.30 ± 14.94	0.00–147.87	11.59 ± 12.86	0.00–95.08	0.022
Vitamin A (μg/day)	729.09 ± 616.38	138.87–6409.44	613.61 ± 624.87	54.08–6897.98	620.11 ± 510.58	88.33–5034.54	0.059
b-Carotene (μg/day)	1232.07 ± 615.54	137.83–3812.99	1300.97 ± 762.26	126.20–6014.49	1421.19 ± 811.94	176.36–5889.25	0.027
Thiamin (vitamin B1) (mg/day)	1.06 ± 0.35	0.37–2.36	0.99 ± 0.35	0.28–3.08	1.05 ± 0.36	0.30–2.24	0.053
Riboflavin (vitamin B2) (mg/day)	1.31 ± 0.43	0.50–3.15	1.24 ± 0.48	0.29–3.27	1.36 ± 0.51	0.31–3.46	0.018
Niacin (vitamin B3) (mg/day)	19.18 ± 6.34	5.70–39.98	17.58 ± 5.95	3.83–42.51	18.81 ± 6.10	3.93–37.39	0.010
Vitamin B6 (mg/day)	1.75 ± 0.59	0.44–3.66	1.67 ± 0.55	0.49–4.34	1.78 ± 0.61	0.63–3.51	0.096
Folate (μg/day)	209.16 ± 64.94	49.56–443.19	199.87 ± 65.40	59.57–409.63	230.98 ± 77.57	73.80–499.30	<0.001
Vitamin B12 (μg/day)	4.64 ± 2.30	1.30–15.72	4.26 ± 1.95	0.10–14.45	4.49 ± 2.08	0.79–13.55	0.133
Vitamin C (mg/day)	85.10 ± 41.11	13.44–282.00	88.09 ± 44.93	11.08–303.30	92.54 ± 45.99	11.23–401.16	0.215
Vitamin D (μg/day)	4.13 ± 1.75	1.32–11.83	3.73 ± 1.60	0.53–9.43	3.85 ± 1.66	0.49–10.06	0.025
Vitamin E (mg/day)	14.82 ± 5.72	5.15–41.35	13.38 ± 5.07	3.36–33.46	14.85 ± 5.50	6.16–40.28	0.003
Iron (mg/day)	11.03 ± 3.26	4.11–26.03	10.05 ± 3.13	3.38–19.88	11.14 ± 3.34	3.78–21.15	<0.001
Magnesium (mg/day)	331.02 ± 100.86	99.65–713.50	308.30 ± 98.16	118.17–710.15	357.36 ± 118.98	117.56–829.09	<0.001
Selenium (mg/day)	0.05 ± 0.02	0.02–0.11	0.04 ± 0.02	0.01–0.10	0.05 ± 0.02	0.01–0.10	0.107
Zinc (mg/day)	10.13 ± 3.23	3.85–23.60	9.35 ± 2.85	2.51–20.63	10.40 ± 3.20	3.59–20.03	0.001
Tea (g/day)	248.06 ± 318.31	0.00–1625.00	270.67 ± 319.29	0.00–1950.00	260.62 ± 319.02	0.00–1950.00	0.731
ADII	0.052 ± 2.41	−9.02–7.64	0.055 ± 2.47	−9.03–6.20	0.054 ± 2.33	−9.74–4.93	>0.999

IBD = inflammatory bowel disease; IBS = irritable bowel syndrome; HC = healthy controls; ADII = Adapted Dietary Inflammatory Index. Continuous data expressed as mean ± standard deviation (SD). Difference between IBD, IBS and HC was tested with ANOVA and post-hoc Bonferroni correction. More details on the ADII can be found in Van Woudenberg et al. [13].

## Appendix B

The methods and results from the URF analysis can be found in Appendix B, including Figure A1: Principle coordinate analysis (PCoA) score plot based on food products; Figure A2: Principle coordinate analysis (PCoA) score plot based on nutrients—PCo1 and PCo2; Figure A3: Principle coordinate analysis (PCoA) score plot based on nutrients—PCo4 and PCo7.

### Appendix B.1. Methods

In addition to using predefined indices (i.e., Dutch Healthy Diet Index 2015 and Adapted Dietary Inflammatory Index), an explorative unsupervised random forest (URF) analysis [36] was performed to investigate the combined effects of sets of food items and nutrients as potential differentiating factors between inflammatory bowel disease (IBD) patients, irritable bowel syndrome (IBS) patients and healthy controls (HC). This unsupervised machine learning technique allows for an investigation of the natural grouping that occurs in the data based on the input variables. Consequently, the outcome can be visualised using a principal coordinate analysis (PCoA) score plot. In this plot, each point represents a single participant. Individuals are colour-coded according to the investigated groups, i.e., patients in remission, IBD patients with active disease, IBS patients and HC. In this study, the URF was performed on two sets of variables (food items and nutrients) derived from the food frequency questionnaires.

### Appendix B.2. Results

In the PCoA score plot based on food products (Figure A1), no clear separation was observed for the investigated groups. Similarly, the PCoA score plot based on the nutrients (Figure A2) did not show any groupings when the first PCos were considered. However, when PCo4 and PCo7 were considered (Figure A3), IBS patients were clearly separated from HC and IBD along PCo4. It is relevant to mention that PCo4 and PCo7 describe only a small portion of the total variance (less than 4%). This suggests that although the differences between the groups of interest are there and they correspond to the differences with the nutrients’ level, the small amount of variance describing those differences indicate minor alterations between IBS and the rest based on the nutrient variables. The most important set of variables that cause those differences were in majority reduced in IBS individuals.
