# Supplementary material for: Diet Quality and Dietary Inflammatory Index in Dutch Inflammatory Bowel Disease and Irritable Bowel Syndrome Patients
Source: Nutrients. 2022 May 6;14(9):1945. doi: 10.3390/nu14091945 (PMC9101333; doi:10.3390/nu14091945)
Supplement: Supplementary file 1 [file nutrients-14-01945-s001.zip › nutrients-1686830-supplementary.pdf]

Supplementary Materials

Table S1. Dietary indices, intestinal inflammation and gastrointestinal symptoms per disease activity status for inflammatory bowel disease.

	**Remission** (n = 158)		**Active disease** (n = 80)		**p-Value**
DHD-2015	71.15 ± 16.72	(n = 158)	64.74 ± 15.38	(n = 80)	0.004
ADII	−0.055 ± 2.107	(n = 158)	0.263 ± 2.932	(n = 80)	0.390
Calprotectin (µg/g)	42.6 ± 50.7	(n = 137)	491.5 ± 627.4	(n = 72)	<0.001
GSRS					
Abdominal pain	2.0 ± 0.9	(n = 55)	2.3 ± 1.1	(n = 25)	0.097
Constipation syndrome	1.7 ± 1.0	(n = 52)	2.2 ± 1.1	(n = 25)	0.102
Diarrhoea syndrome	2.4 ± 1.4	(n = 53)	3.3 ± 1.5	(n = 24)	0.013
Indigestion syndrome	2.6 ± 1.0	(n = 55)	3.1 ± 1.5	(n = 25)	0.193
Reflux syndrome	1.4 ± 0.7	(n = 55)	1.5 ± 0.8	(n = 25)	0.525

DHD-2015 = Dutch Healthy Diet index 2015; ADII = Adapted Dietary Inflammatory Index; GSRS = Gastrointestinal Symptom Rating Scale.

Continuous data expressed as mean ± standard deviation (SD). Difference between phenotypes was tested with two-sample t-test.

Table S2. Dietary indices, intestinal inflammation and gastrointestinal symptoms per inflammatory bowel disease phenotype.

	**Crohn's disease** (n = 156)		**Ulcerative colitis** (n = 82)		**p-Value**
DHD-2015	65.47 ± 15.94	(n = 156)	75.71 ± 15.61	(n = 82)	<0.001
ADII	0.193 ± 2.53	(n = 156)	−0.217 ± 2.18	(n = 82)	0.214
Calprotectin (µg/g)	199.3 ± 411.5	(n = 136)	193.4 ± 455.6	(n = 73)	0.924
GSRS					
Abdominal pain	2.1 ± 0.9	(n = 51)	2.0 ± 1.0	(n = 29)	0.507
Constipation syndrome	2.0 ± 1.2	(n = 48)	1.6 ± 0.8	(n = 29)	0.053
Diarrhoea syndrome	2.9 ± 1.6	(n = 48)	2.3 ± 1.2	(n = 29)	0.047
Indigestion syndrome	2.8 ± 1.2	(n = 51)	2.6 ± 1.2	(n = 29)	0.487
Reflux syndrome	1.5 ± 0.9	(n = 51)	1.3 ± 0.6	(n = 29)	0.272

DHD-2015 = Dutch Healthy Diet index 2015; ADII = Adapted Dietary Inflammatory Index; GSRS = Gastrointestinal Symptom Rating Scale.

Continuous data expressed as mean ± standard deviation (SD). Difference between phenotypes was tested with two-sample t-test.

Table S3. Intestinal inflammation and gastrointestinal symptoms per irritable bowel syndrome subtype.

	IBS-C (n = 56)		IBS-D (n = 93)		IBS-M (n = 103)		p-Value
DHD-2015	73.39 ± 15.41	(n = 56)	72.41 ± 17.30	(n = 93)	70.53 ± 16.76	(n = 103)	0.541
ADII	−0.133 ± 2.483	(n = 56)	0.142 ± 2.770	(n = 93)	0.113 ± 2.221	(n = 103)	0.786
Calprotectin (µg/g)	59.0 ± 56.5	(n = 17)	46.3 ± 61.8	(n = 33)	85.9 ± 114.1	(n = 37)	0.163
GSRS							
Abdominal pain	3.4 ± 1.1	(n = 56)	3.3 ± 1.3	(n = 92)	3.4 ± 1.3	(n = 101)	0.801
Constipation syndrome	4.4 ± 1.3	(n = 56)	2.6 ± 1.0	(n = 91)	3.5 ± 1.2	(n = 101)	<0.001
Diarrhoea syndrome	2.2 ± 1.1	(n = 56)	3.9 ± 1.5	(n = 91)	3.5 ± 1.4	(n = 102)	<0.001
Indigestion syndrome	4.2 ± 1.2	(n = 55)	4.1 ± 1.4	(n = 92)	4.2 ± 1.3	(n = 101)	0.994
Reflux syndrome	2.4 ± 1.5	(n = 56)	2.1 ± 1.3	(n = 92)	2.2 ± 1.4	(n = 101)	0.321

IBS = irritable bowel syndrome; IBS-C = constipation predominant IBS; IBS-D = diarrhoea predominant IBS; IBS-M = mixed stool pattern IBS; DHD-2015 = Dutch Healthy Diet index 2015; ADII = Adapted Dietary Inflammatory Index; GSRS = Gastrointestinal Symptom Rating Scale.

Difference between subtypes was tested with ANOVA and post-hoc Bonferroni correction. Unspecified subtype IBS (IBS-U) was not included in this comparison due to the small sample size (n = 9).
